# Acute arterial embolism of left lower extremity caused by paradoxical embolism in Ebstein's anomaly: A case report

**DOI:** 10.1097/MD.0000000000005901

**Published:** 2017-02-03

**Authors:** Jun-Sheng LI, Jie Ma, Zi-Xing Yan, Dong-Ming Cheng, Liang Chang, Hai-Chun Zhang, Jiang-Yan Liu

**Affiliations:** a Shanxi Medical University, Taiyuan, Shanxi; b Department of Cardiothoracic Surgery; c Department of Ultrasound, Shanxi Medical University Second Hospital, Taiyuan, Shanxi, P.R. China.

**Keywords:** amputation, cone reconstruction, Ebstein's anomaly, gangrene, paradoxical embolisms

## Abstract

**Introduction:**

Ebstein's anomaly is a benign and stable congenital heart disease for asymptomatic patients. Despite a low incidence of Ebstein's anomaly (EA), patients’ quality of life can be badly affected by EA without positive surgical intervention. Especially EA is associated with other congenital heart disease, such as the atrial septal defect, patent foramen ovale, and arterial embolism exclude other reasons, it is often considered to be the consequence of paradoxical embolism, and surgical intervention must be conducted.

**Case report:**

An 11-year-old girl falling off the bed suffered pain from left lower extremity. Echocardiographic evaluation revealed an EA, severe tricuspid regurgitation, and secundum atrial septal defect. Both left leg amputation and cardiac surgery were conducted after recovery. Under the condition of anesthesia cardiopulmonary bypass extracorporeal circulation, atrial septal defect repair and Cone reconstruction of the tricuspid valve were performed. Patient recovered well and left hospital smoothly.

**Discussion:**

EA is a rare and complex congenital cardiac malformation. There are about 80% to 90% of EA patients with combined atrial septal defect and patent foramen ovale. Sudden arterial occlusion is very rare especially in childhood. When thoracic roentgenoscopy, arterial blood gas analysis, coagulation test, and echocardiographic of lower extremity deep venous system are all normal, one should consider the possibility of a paradoxical embolism. If patients have the paradoxical embolism or worsening tricuspid regurgitation, the most suitable therapeutic regimen should be chosen according to patients’ condition. With surgical techniques and methods renewed continuously, cone reconstruction of the tricuspid valve has been confirmed in clinical trials, which can use its own tissues to form not only central bloodstream, but also the coaption between leaflet and leaflet.

## 1 Introduction

EA is a benign and stable congenital heart disease for asymptomatic patients. When combined atrial septal defect, patent foramen ovale, arterial embolism and other reasons are all excluded, it is often considered to be the consequence of paradoxical embolism, and surgical intervention must be conducted. In order to provide guarantee for the quality of life and relieve the pain of patients and their families, early diagnosis and treatment can avoid the occurrence of serious complications. Cone reconstruction is suitable for most patients, especially for newborns and young people under the age of 18, which can make up for shortcomings and deficiencies along with patients’ growth in other surgical methods. Cone reconstruction of the tricuspid valve is the latest surgical treatment, which can use its own tissues to form not only central bloodstream, but also the coaption between the leaflet and the leaflet. Literatures reported that the majority of patients’ tricuspid valve kept a good function of postoperative .

## 2 Case presentation

**Figure 1:**
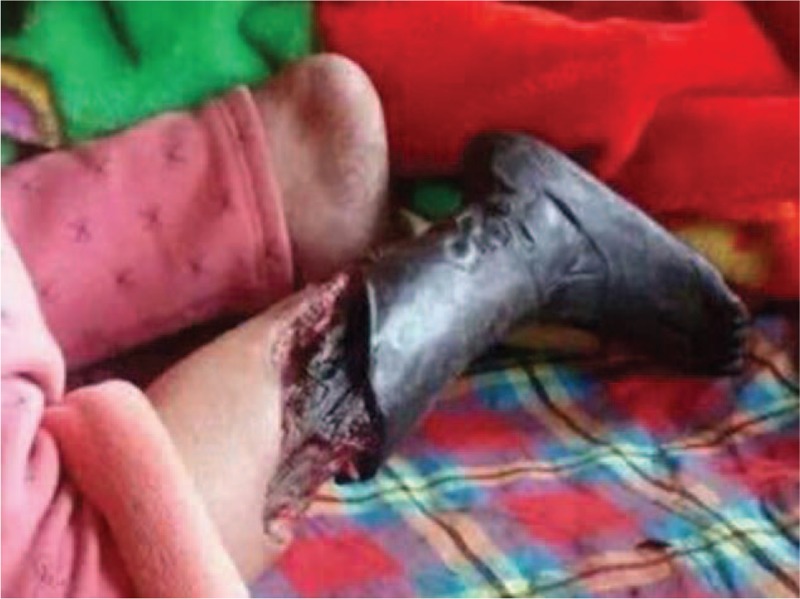
An ischemic appearance and gangrene of left lower extremity. 2/3 anterior lateral bones are exposed in the middle tibia.

**Figure 2:**
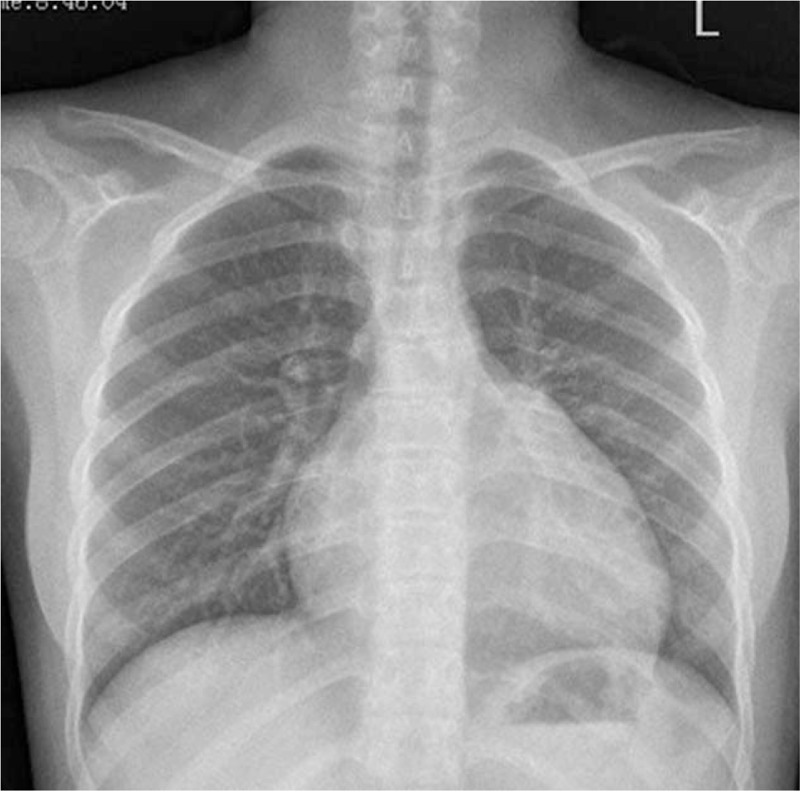
Chest radiograph showed enlargment of cardiac shadow and the cardiothoracic ratio was 0.6.

**Figure 3:**
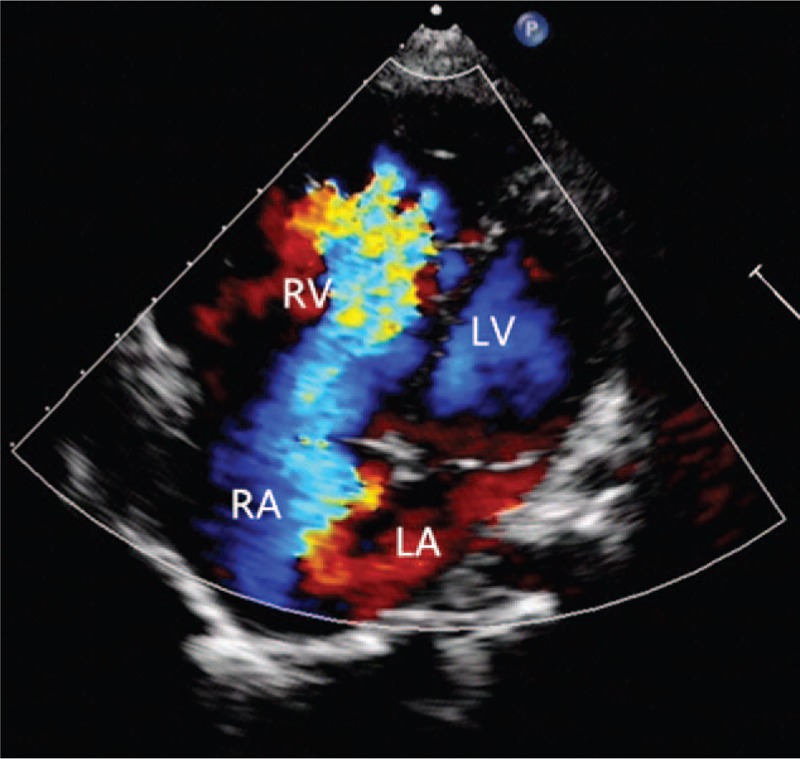
Echocardiographic image in the apical 4-chamber view demonstrated systolic severe tricuspid regurgitation.

**Figure 4:**
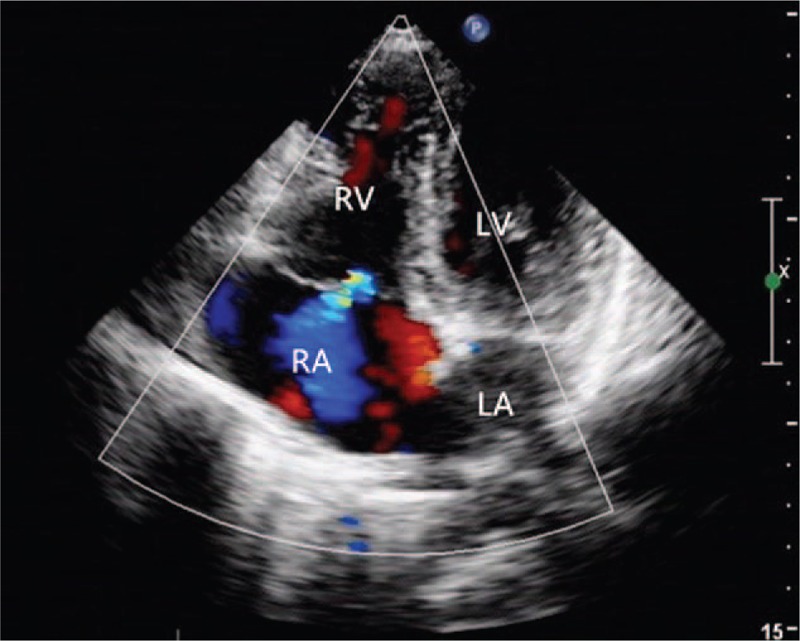
Echocardiographic image in the apical 4-chamber view showed slight of tricuspid regurgitation.

An 11-year-old girl falling off the bed suffered pain from left lower extremity. She was admitted to the local hospital with left lower extremity gangrene, and then she was transferred to our hospital. Physical examination: the skin of left calf knee joints was blackened and dried; in the middle tibia, 2/3 anterior lateral bones were exposed with stench; the left knee joint was movable and left ankle joint had activity disorders, in addition, the left knee joint showed skin temperature differences (Fig. 1) . Blood pressure 116/64 mm Hg, heart 118 bpm, stable cardiac rhythm. A blowing systolic murmur 2/6 presented between the first to second left intercostal sternal border and a systolic murmur 1/6 located at tricuspid valve. Other physical examinations were normal. Thoracic roentgenoscopy (Fig. 2) showed the dilation of RV and increased pulmonary arterial pressure and the cardiothoracic ratio was 0.6. Echocardiographic evaluation (Fig. 3) revealed an EA, severe tricuspid regurgitation, and secundum atrial septal defect. Lower-extremity doppler-imaging showed left lower extremity arterial occlusion (the possibility of thrombembolia). Laboratory examination demonstrated a normal coagulation test with oxygen saturation 98% in arterial blood gas analysis. Left leg amputation was conducted on that day. Cardiac surgery was conducted after recovery. Before the operation, the patient was given the medical treatments with cardiotonic, diuretic and vasodilator effects. Under the condition of anesthesia cardiopulmonary bypass extracorporeal circulation, atrial septal defect repair and cone reconstruction of the tricuspid valve were performed. Patient recovered well and left hospital smoothly. Nine months after the operation, echocardiographic showed slight tricuspid regurgitation (Fig. 4) .

## 3 Discussion

EA^[1]^ is a rare and complex congenital cardiac malformation,^[2,3]^ which was initially founded by Breslaus doctor Wilhelm Ebstein in 1866 when he did the heart autopsy. EA accounts for 0.5% to 1% of all congenital cardiac disease,^[4–7]^ and only 5% of patients survive beyond the fifth decade.^[8,9]^ The pathological changes of EA are that septal, posterior and anterior leaflet (in few cases) of tricuspid valve are not adhere to tricuspid annulus which move down to RV spirally with textural anomalies and paramorphia of atrioventricular sulcus, valve, flap structure, and RV.^[10,11]^ Because of interactivity of RV agenesis and dysfunction, together with tricuspid incompetence, RV has an overload of volume and cardiac morphology increased, as a result, anoxia, heart failure, and arrhythmia can be the threat to life,^[12]^ but complications associated with external cardiac first symptoms are more serious, including cerebrovascular accident, brain abscess, and peripheral arterial embolization, which brings heavy strike to patients and families. Therefore, early surgical correction is the most effective therapeutic method to cure tricuspid regurgitation, abnormal ventricular movement, and other combined malformation.

Sudden arterial occlusion is very rare especially in childhood and it usually occurs at artery bifurcation, which mainly threatens affected extremity or patient survival. In spite of extensive collateralization, the level of embolism and length of time of onset to treatment are major factors that influence limbs survival.^[12]^ When thoracic roentgenoscopy, arterial blood gas analysis, coagulation test, and echocardiographic of lower extremity deep venous system are all normal, one should consider the possibility of a paradoxical embolism.^[13–15]^ Paradoxical embolism is a systemic arterial embolism caused by the abnormal passage of a venous thromboembolus through a right-to-left shunt, which is considered to be the major cause that results in ischemic events in adult.^[16]^ In general, inference and clinical fallot are diagnostic criteria^[17,18]^: (1) There is no obvious thrombus source of arterial circulation system; (2) deep vein thrombosis or pulmonary embolism; (3) there exit reversed or reversible shunt of heart defect; (4) there exit persistent (primary or secondary pulmonary hypertension) or transient (valsalva maneuver or cough) gradient pressure that can promote right to left shunt.

There are about 80% to 90% of EA patients with combined atrial septal defect and patent foramen ovale.^[4,19]^ Although paradoxical embolism such as stroke, brain abscess, and peripheral artery are rare,^[20,21,22]^ if patients have the paradoxical embolism or worsening tricuspid regurgitation, the most suitable therapeutic regimen should be chosen according to patients’ condition.^[17,23]^ Echocardiogram presents that the patient has bidirectional blood flow through the atrial septal defect, but cough, sneeze, squat fast, emiction, and cacation can make right atrial pressure increase transiently. The patient has the medical history of falling off the bed. In addition, both mild pulmonary hypertension and increased right atrial pressure predispose for transient right to left shunt and paradoxical embolization.^[22,24]^ In the presence of atrial septal defect, increasing right atrial pressure may cause changes of hemodynamics and function and right to left reverse shunt which is the anatomical basis of paradoxical embolism resulting in arterial embolism.^[25]^ In 2014, Christine H put forward a possible paradoxical embolic event (PPEE), which was defined as the history of transient ischemic events such as cerebrovascular accident, brain abscess or an event assumed to be embolic without another explanation. In the absence of pathologic confirmation, these events were assumed to be the paradoxical embolism events.^[26]^

The patient has a history of arterial embolism and severe tricuspid regurgitation, surgical indications are clear.^[23]^ In consideration of age and postoperative prognosis effect in long term, cone reconstruction of the tricuspid valve associated with closure of secundum atrial septal defect was conducted on patient after the patient's condition was stable(after amputation), trying to avoid the occurrence of reoperation and valve replacement.^[27,28]^ Cone reconstruction of the tricuspid valve is the latest surgical treatment, which can use its own tissues to form not only central bloodstream, but also the coaption between leaflet and leaflet. Literatures reported that the majority of patients’ tricuspid valve kept a good function of postoperative.^[28–32]^ There are 3 elements of the operation points^[33–35]^: first, reduce the dilated tricuspid annulus to normal size; second, dissociate all leaflets by excising the secondary chordae tendineae and abnormal tissue that limit leaflets movement; third, reconstruct a cone structure of tricuspid valve by suturing junction. In treatment of EA with modified Carpentier's method in early time, the surgeon used autologous pericardial to reinforce tricuspid annulus, which got good effects.^[36]^ Cone reconstruction of the tricuspid valve still use this way. For patients, advantages of using autologous pericardial to reinforce reconstructive tricuspid annulus are as below: (1) it is easy for endocardium cells to stretch, grow and shrink tricuspid annulus, which reinforce tricuspid annulus; (2) compliance of tricuspid annulus is not affected; (3) there is no need for anticoagulation, which can avoid the occurrence of postoperative thromboembolism; (4) prevent the reoperation because of occurrence of tricuspid regurgitation after operation at the junction of the posterior septal suture. Moreover, some key points should be paid more attention in operation. (1) Management techniques for parietal septum and dysplasia and downward displacement malformation of parietal septum. Reconstructing new tricuspid valve needs to transfer proximal end of parietal septum with downward displacement malformation generally and to debond connection between interventricular septum. If it is too short, when it cannot reach real tricuspid annulus, it folds proximal edge of parietal septum toward tricuspid annulus center and extend parietal septum longitudinally. Then, a wider and bigger conical structure can be formed by suturing leading edge of parietal septum and anterior septal margin and fixing posterior leaflet free margin and the other side of anterior leaflet. (2) Avoid the occurrence of arrhythmias. New tricuspid annulus is reconstructed in normal anatomical position; folding real tricuspid annulus is to match with reconstructed conical valve. To avoid block and severe ventricular arrhythmias is to suture superficially near the atrioventricular node. (3) Handle the combined with other cardiac malformations. On the closure of atrial septal defect, there is a right to left shunt that must be retained; for patent foramen ovale, valve closure can be formed by suturing; if interauricular septum is complete in operation, it should be opened along the inclined edge of the oval fossa; if it is secundum atrial septal defect, closure of valve technology should be taken. For patients with Ebstein's anomaly combined atrial septal defect, closure atrial septal defect is not recommend, but the closure atrial septal defect is surgical indication if there is a history of paradoxical embolism.^[23]^

However selection of surgical indications and timing of surgery depend on patient s’ onset age, malformation type, development of valve and if there are other cardiac malformations and complications such as initial symptoms except for symptoms that are related to heart and cardiac function. All above these should be taken into overall consideration. Cone reconstruction of the tricuspid valve is an effective measure to treat Ebstein's anomaly.
